# 

**Published:** 1895-11-02

**Authors:** 


**Notices of Births, Deaths, and Marriages** are inserted in this column at a charge of 2s. 6d. for an announcement not exceeding four lines.

**Notice to Advertisers and Subscribers.**

All Advertisements, Orders for Copies of The Hospital and Business Communications should be addressed to **THE MANAGER, (Not to The Editor)**, The Hospital, 428, Strand, London, W.C.

The Cost of Subscription, post free, is as follows:—Per week, 2½d.; per quarter, 2s. 8d.; per half-year, 5s. 3d.; per annum, 10s. 6d. Foreign:—Per Annum, 15s. 2d.; payable, in all cases, in advance.

### NOTICE TO CORRESPONDENTS.

All MS., Letters, Books for Review, and other matters intended for the Editor should be addressed The Editor, The Lodge, Porchester Square, London, W.

The Editor cannot undertake to return rejected MS., even when accompanied by stamped directed envelope.

**"Burdett's Hospital Annual," 1895,**

Sixth year of publication. 950 pp. Scarlet cloth, gilt lettered. Price 5s. A most complete guide to British, Colonial, Indian, and American Hospitals, Dispensaries, Nursing and Convalescent Institutions, and Asylums. A few complete sets of the preceding five issues of the "Annual" may still be obtained on application to the Publishers, The Scientific Press (Limited), 428, Strand, London, W.C.

**NOTICE.—Vol. XVIII. of "The Hospital" (April to September, 1895), in handsome cloth binding, is now on sale at the Office of this Journal. Price 6s. Cases for binding the Numbers contained in this Volume, 1s. 6d. Index to Vol. XVIII., 2½d., post free.**

**The Monthly Part for November is now ready, price 6d.; by post, 8d.**

### PICRIC ACID AS A CURE FOR BURNS.

Dr. Thierry, physician to the Hospice de Charité, Paris, would appear to have discovered a new and very remarkable cure for burns in the form of a saturated solution of picric acid, which, if applied to small burns and scalds, removes pain and achieves a singularly speedy cure. The experiments which have induced Dr. Thierry to put forward this new remedy originated in himself having more than once, when using picric acid disinfectant, spilled burning substances upon his hands with surprisingly harmless results, leading him to the conclusion that the presence of the acid might account for the immunity. Such a discovery, if accredited by further experience, will confer an immense boon upon humanity, seeing the terrible amount of suffering yearly inflicted through accidents from burning. The application of the solution is said to cause a temporary yellow discoloration of the skin, but this can be removed by the use of boric acid.

disease. Such an institution for the benefit of the large and struggling class above the very poor, for whom many charities already exist, has been much needed, and Mr. Yarrow's endeavour to meet this need should meet with ready recognition and appreciation. Full particulars with regard to terms of admission may be had from the Secretary, at the London office, 73A, Queen Victoria Street.

### AROUND THE HOSPITALS.

### SCRAPS AND GLEANINGS.

THE hospital demonstration at Saltburn brought in £20 to the funds of the North Ormesby Cottage Hospital.

THE Mayor of Stratford-on-Avon has given £50 towards the liquidation of the debt existing on the local hospital.

A LEGACY of £20 has been bequeathed by Miss Harriet Read to the Dublin National Eye and Ear Infirmary.

THE trustees of the "Denny Gift Fund" have presented a sum of £50 to the funds of the Dumbarton Eye Dispensary.

A HARVEST thanksgiving service was held last week at the National Hospital for the Paralysed and Epileptic, Bloomsbury.

DURING the existence of the Burton Infirmary Saturday Collection, £15,754 has been collected for the funds of the Infirmary.

THE Ladies' Committee of the Hull Saturday Fund has just had the annual meeting; the balance sheet is of a satisfactory nature.

THE Plymouth Public Dispensary has, during the past year, issued 3,481 tickets of recommendation to the sick and deserving poor of the neighbourhood.

WE are glad to note a small increase in the funds of the Salford Royal Hospital, Manchester, but the adverse balance at the bank is standing at over £1,000.

THE collection in the elementary schools and neighbourhood towards the maintenance fund of the Convalescent Home at Weston-super-Mare has reached £166.

ONE of the most munificent of the original donors to the Pasteur Institute in Paris was the late Tsar Alexander III., who gave as a donation 100,000 francs.

A CONVERSAZIONE and Cinderella dance was held last week in the Rotunda Hospital, Dublin, in aid of funds for the complete equipment of the new wards and building.

A PAPER entitled "Befriending the Friendless Girl," by the Marquis of Lorne, will appear in the November part of the "Quiver," which commences the new volume.

THE weather was sadly against the Hospital Street Collection made at Ipswich in favour of the funds of the East Suffolk Hospital, and seriously affected the takings.

THE growing demand for nurses from the Royal Portsmouth Hospital has made it necessary for the committee to further extend the premises in order to receive a greater number.

THE Lincoln County Hospital is not flourishing financially. At the close of the year it is conjectured the adverse balance against the banking account will have reached £1,000.

THE total amount of the new building fund of the Bedford Infirmary amount is £21,622, the subscribers numbering 400. The House of Russell gave £4,777, and Mr. S. Whitbread £5,000.

A COUNTY COUNCIL inquiry has been held at Lytham into the advisability of establishing a joint hospital board for the Fylde district, with regard to the isolation of infectious diseases.

THE late Mrs. Alexander devoted the profits from the sales and copyright fee derived from granting permission to reprint her hymns and poems to the Asylum for Deaf Mutes in Ireland.

THE nurses of the Rotunda Hospital, Dublin, have collected a sum of £25 towards furnishing the new wing at the institution. Mr. S. Adair has sent a donation of £25 with the same object.

THE Mercers' Hospital, at Dublin, is much in need of funds. This hospital fulfils a useful purpose, every day there are not less than 25 accident cases treated, 40 dispensary, and a large number of intern patients.

AMONG quite recent additions to St. George's Hospital Medical School is the establishment of a prize for proficiency in bacteriology. This has been established through the instrumentality of Mrs. F. Webb, who has endowed it with the sum of £1,000.

A MEETING was lately convened in protest against the furtherance of the present scheme of the Hastings Isolation Hospital, the proposed site being opposed by the ratepayers of Battle, in which locality it is suggested the hospital shall be placed.

MRS. BENSON, wife of the Archbishop of Canterbury, visited Weymouth last week in order to open a home of rest for women and girls which has been purchased and furnished at a cost of £2,000, in memory of the late Mrs. Wordsworth, wife of the Bishop of Salisbury.

THE finances of the Batley Cottage Hospital, Dewsbury, are in a satisfactory condition, and the institution is now on a good working basis, and in many ways it has amply justified its claims for the sympathy and generous support which it receives from all classes of the community.

IN consequence of the great increase in the number of patients at the Dudley Dispensary a qualified dispenser has been appointed at an increase of £52 to the expenditure. A sub-committee has been appointed to take into consideration various proposals for the removal of the incubus of debt existing on the institution.

THE Whitchurch Cottage Hospital presents a highly-praiseworthy report this year. We note that not only were the number of patients in excess of previous records, but that there is a balance to the good on the account sheets. The committee, however, are of opinion that the operation of the hospital as a central nursing institution for the neighbourhood admits of considerable expansion, to which effect will be given as opportunity occurs.

A WAR of protest continues to rage in Dublin against the erection of the proposed new infectious diseases hospital. The wildest objurgations have been cast upon the scheme, to which, last week, the Chairman of the Public Health Committee vigorously replied. The essential and main objects of this scheme are to prevent small-pox being treated within the City of Dublin, and the provision of a onvalescent home for fever cases.

### The Student of Medicine.

### APPOINTMENTS.

H. Bennett, M.D.Edin., F.R.C.P.Lond., appointed Consulting Physician to the Hospital for Epilepsy and Paralysis, Regent's Park. B. A. Bottomley, M.B., B.C.Cantab., appointed Senior Resident Medical Officer to the Royal Free Hospital. J. A. Craig, M.B., B Ch.R.U.I., appointed Second House Surgeon to the Royal Southern Hospital, Liverpool. Dr. N. F. Edwards, appointed House Physician to the Swansea Hospital. A. Emson, L.R.C.P., L.R.C.S.Edin., appointed Medical Officer for the Workhouse and Dorchester District of the Dorchester Union. Mr. E. P. Evall, appointed Medical Officer for the Waterbeach District of the Chesterton Union. L. Guthrie, M.A., M.B.Oxon., appointed to the charge of In-patients, in addition to that of Out-patients, at the Hospital for Epilepsy and Paralysis, Regent's Park. Dr. G. Higginson, appointed Medical Officer of the Workhouse and the First District of the Church Stretton Union. Dr. Kidd, appointed Resident Medical Officer of the Dundee Hospital for the Sick Poor. C. Lamplough, M.R.C.S., L.R.C.P.Lond., appointed Resident Clinical Assistant to the St. Marylebone Infirmary, Notting Hill. D. L. Lindsay, L.R.C.P. and S.Edin., L.F.P.S.Glasg., appointed House Physician to the Glasgow Royal Infirmary. R. S. Martin, L.R.C.P., L.R.C.S.Edin., L.F.P. and S. and L.M.Glasg., appointed Medical Officer and Public Vaccinator for the Lever District of the Bolton Union. S. Poole, M.B., C.M.Edin., appointed Medical Officer for the Second Wolverhampton District of the Wolverhampton Union. C. H. Powers, L.R.C.P.Lond., M.R.C.S.Eng., appointed Medical Officer for the Gosforth District of the Whitehaven Union. F. G. Proudfoot, M.A.St.And., M.B., C.M.Edin., appointed House Surgeon in the Radcliffe Infirmary, Oxford. W. B. Roué, M.D., M.S., appointed Consulting Physician to the Bristol Hospital for Sick Children and Women. W. H. Steele, M.B., C.M.Edin., appointed House Surgeon to the Lancaster Infirmary. T. W. Thomas, M.R.C.S.Eng., L.S.A., appointed Medical Officer for the Caerphilly District of the Pontypridd Union. J. R. Thompson, M.B., B.Ch.R.U.I., appointed Third House Surgeon to the Royal Southern Hospital, Liverpool. J. L. Watt, M.A.Aberd., M.B., C.M., appointed Medical Officer for the Second Division of the Third District of the St. Germans Union.

### THE STUDENT OF MEDICINE—*continued.*

### VACANCIES.

The following vacancies are announced this week, the amount of salary in each case being given after the name of the institution:—

#### Resident Medical Officers.

Birmingham and Midland Free Hospital for Sick Children.—Resident Medical Officer and Resident Surgical Officer. Salaries £70 and £50 respectively, with board, washing, and attendance at the institution. Applications to the Secretary, Children's Hospital, Steelhouse Lane, Birmingham, by November 5th.

Bristol Hospital for Sick Women and Children.—House Surgeon; doubly qualified. Salary £100 per annum, with rooms and attendance (not board). Applications and testimonials, endorsed "House Surgeon," to H. Lawford Jones, Secretary, before November 6th.

Liverpool Eye and Ear Infirmary.—House Surgeon; doubly qualified. Salary £80, with residence and maintenance. Applications to R. Haigh, Hon. Sec., Berey's Buildings, George Street, Liverpool, by November 2nd.

Sunderland Infirmary.—House Physician. Salary £80, rising £10 annually to £100, with board and residence. Applications to the Chairman of the Medical Board by November 7th.

Whitehaven and West Cumberland Infirmary.—House Surgeon; doubly qualified. Salary £120 per annum, and £30 per annum for dispensing, with furnished apartments and attendance. Applications and testimonials to Tyson Kitchen, Secretary, by November 9th.

#### Non-Resident Medical Officers.

Belgrave Hospital for Children, 77 and 79, Gloucester Street, S.W.—Surgeon to Out-patients; must be F.R.C.S.Eng. Applications to the Honorary Secretary by November 2nd.

Brown Animal Sanatory Institution.—Professor Superintendent. Salary £250 per annum. Applications to the Registrar of the University of London, Burlington Gardens, W., by November 15th.

City of Dublin Hospital.—Visiting Surgeon. Applications to Mr. Arthur Benson, F.R.C.S.I., Hon. Sec., Medical Board, City of Dublin Hospital, Upper Baggot Street, Dublin, before November 5th.

Glasgow Maternity Hospital.—Obstetric Physician and Assistant Obstetric Physician. Applications to Arthur Forbes, Secretary, 146, Buchanan Street, Glasgow, by November 8th.

Royal Berkshire Hospital.—Consulting Dentist; must be registered Licentiate in Dental Surgery. Applications to the Secretary ten days before the election on November 5th.

St. Pancras and Northern Dispensary, 126, Euston Road, N.W.—Honorary Surgeon. Applications to H. Peter Bodkin, Hon. Sec., 23, Gordon Street, Gordon Square, W.C., by November 2nd.

Stockton and Thornaby Hospital, Stockton-on-Tees. House Surgeon; doubly qualified. Must reside near the hospital, and devote the whole of his time to the institution. Salary £200 per annum. Applications and testimonials to H. G. Sanderson, Secretary, by November 5th.

### UNIVERSITIES AND COLLEGES.

#### University of Cambridge.

Botany.—The election to the Professorship of Botany, vacated by the death of Professor Babington, will take place on Saturday, November 2nd, at 2.30 p.m.

Physics.—Mr. C. T. R. Wilson, of Sydney, has been appointed Assistant Demonstrator at the Cavendish Laboratory. A Clerk-Maxwell Studentship in Physics, vacant by the resignation of Mr. Whethams, will be filled up this term. The studentship is worth £180 a year for three years.

Pharmacology and Therapeutics.—Professor Bradbury announces a class in these subjects this term, at the Downing Laboratory and Museum, for students preparing for the Third M.B.Camb., and the Intermediate M.B.Lond.

Degree.—At the Congregation, on October 10th, George Pinder, of Queens' College, was admitted to the degree of M.D.

Fellowship.—Mr. Ivor Lloyd Tuckett, B.A., First Class in Human Anatomy and Physiology in the Natural Sciences Tripos of 1894, has been elected to a Fellowship at Trinity College.

#### University of Edinburgh.

The following candidates have passed the First Professional Examination for the degrees of M.B. and Ch.B. in the subjects prefix ed to their names:—

Chemistry.—R. E. Clark, W. Hutcheson, W. J. M McFarlan, A. E. McLeod, C. F. T. Scott, J. Thornhill, D. B. B. Hughes.

Physics.—J. H. C. Orr, J. B. Thorburn.

The following have passed the Medical Preliminary Examination: C. H. Allen, W. L. L. Alston, J. H. Bell, L. N. Biggs, J. H. R. Bowman, J. C. Boyd, G. F. Buist, J. P. Campbell, L. Campbell, K. A. Cappie, G. S. Carey, D. G. Carmichael, C. W. A. Carroll, A. W. S. Christie, W. P. Cormack, P. D. Cremona, T. A. Davies, C. Douglas, W. Eadie, V. M. Ferguson, A. Fleming, Catherine Fraser, J. S. Geikie, E. M. Glanville, J. M. Glasse, T. Gowans, L. S. Sandeman, W. S. Scott, E. T. Selkirk, J. Shields, J. J. Sinclair, B. C. Spence, C. C. Southon, H. M. Speirs, A. Trotter, J. H. Uphan, A. S. Watson, A. G. Watson, W. Watson, G. D. White, F. M. Wigg, G. Wright, J. H. Wrightson, A. M. Wood, A. L. Gurney, H. W. Gush, G. W. Guthrie, N. E. Harding, L. S. Hill, Eleanor J. Hodson, L. G. Hunter, J. P. P. Inglis, J. G. S. Jamieson, E. A. Klein, A. C. Lennie, G. H. Lewis, W. E. Macfarlane, N. F. M'Hardy, D. M'Kinnon, D. S. Macknight, R. M. Matheson, E. C. C. Maunsell, R. C. Monnington, A. L. Nestor, J. G. Peebles, H. G. Pesel, W. G. Porter, L. T. Price, J. B. Primmer, L. C. Rattray, J. S. E. Robertson, A. L. Roxburgh.

### Medical Appointment.

SUNDERLAND INFIRMARY.

Sixth year of publication. 950 pp. Crown 8vo., red cloth, gilt, 5s.

### BURDETT'S HOSPITAL AND CHARITIES ANNUAL:

**Being the Year-Book of Philanthropy, 1895.**

Edited by HENRY C. BURDETT,

Author of "Hospitals and Asylums of the World," "Cottage Hospitals," "Helps in Sickness and to Health," &c., &c.

"Requires no more than the name of its editor to warrant that a subject of great and growing importance is dealt with in a thoroughly satisfactory manner."—*The Times.*

"Still remains the standard work of reference upon all points which relate to charities."—*Daily Telegraph.*

London: The Scientific Press (Limited), 428, Strand, W.C.

### From A. and C. BLACK'S LIST.

**Text-Book of Operative Surgery.** By Dr. TH KOCHER, Professor of Surgery and Director of the Surgical Clinic in the University of Bern. Translated by special permission of the Author from the Second Enlarged and Improved German Edition, by HAROLD J. STILES, M.B., C.M., Senior Demonstrator of Surgery in the University of Edinburgh; Assistant Surgeon, Royal Edinburgh Asylum for Sick Children. Illustrated with 185 cuts in text. Demy 8vo., cloth, price 20s.

**The Senile Heart.** Its Symptoms, Sequelæ, and Treatment. By GEO. W. BALFOUR, M.D., LL.D., Consulting Physician to the Royal Infirmary and to the Royal Public Dispensary, Edinburgh. Crown 8vo., cloth, price 5s.

**Plea for a Simpler Life.** By GEORGE S. KEITH, M.D., F.R.C.P.E. Crown 8vo., cloth, price 2s. 6d.

**Text-Book of General Pathology and Pathological Anatomy.** By Professor RICHARD THOMA, of Dorpat. Translated by ALEX. BRUCE, M.D., Lecturer on Pathology, Surgeons' Hall, Edinburgh. [*Vol. I. in preparation.*

A. AND C. BLACK, Soho Square, London, W.

Now ready, demy 8vo., blue buckram gilt, 3s. 6d., with numerous Illustrations.

### DIET IN SICKNESS AND IN HEALTH.

By MRS. ERNEST HART,

Formerly Student of the Faculty of Medicine, Paris, and of the London School of Medicine for Women. With an Introduction by Sir HENRY THOMPSON, F.R.C.S., M.B.Lond., who says: "Forms a handbook which will not only interest the Dietetic Student, but offer him, within its modest compass, a more complete epitome thereof than any work which has yet come under my notice."

London: THE SCIENTIFIC PRESS (Ltd.), 428, Strand, W.C.

### STANDARD TEXT-BOOKS FOR NURSES.

THE NURSES' DICTIONARY OF MEDICAL TERMS AND NURSING TREATMENT. By HONNOR MORTEN. 2nd Edition. Demy 16mo. Cloth 2s., leather 2s. 6d.

NURSING. By ISABEL ADAMS HAMPTON. 2nd Edition. Crown 8vo. Cloth, 500 pp. 7s. 6d. net.

THE MOTHER'S HELP AND GUIDE TO THE DOMESTIC MANAGEMENT OF CHILDREN. By P. MURRAY BRAIDWOOD, M.D. Crown 8vo. Cloth gilt. 2s. 6d.

ART OF MASSAGE. By A. CREIGHTON HALE. Demy 8vo. Cloth. Illustrated. 6s.

SURGICAL WARD-WORK AND NURSING. By ALEXANDER MILES, M.D. (Edin.), C.M., F.R.C.S.E. Demy 8vo. Cloth. With numerous illustrations. 3s. 6d.

NURSING: ITS THEORY AND PRACTICE. By PERCY G. LEWIS, M.D. New and enlarged edition ready immediately. Crown 8vo. Cloth, 400 pp. Profusely illustrated. 3s. 6d.

HOSPITAL SISTERS AND THEIR DUTIES. By EVA C. E. LUCKES. 3rd Edition. Crown 8vo. Cloth. 2s. 6d.

OPHTHALMIC NURSING. By SIDNEY STEPHENSON, M.B., F.R.C.S.E. Crown 8vo. Cloth. Illustrated. 3s. 6d.

ART OF FEEDING THE INVALID. By a Medical Practitioner and a Lady Professor of Cookery. Demy 8vo. Cloth, 260 pp. 3s. 6d.

OUTLINES OF INSANITY. By FRANCIS H. WALMSLEY, M.D. Demy 8vo. Cloth. 3s. 6d. Popular Edition, stitched, 1s. 6d.

MENTAL NURSING. By WILLIAM HARDING, M.D. 2 d Edition. Demy 8vo. Cloth. 2s. 6d.

London: THE SCIENTIFIC PRESS (Limited), 428, Strand, W.C.

